# Effective Treatments for Abductor Vocal Cord Paralysis: A Comprehensive Review

**DOI:** 10.7759/cureus.67438

**Published:** 2024-08-21

**Authors:** Gowtham Narasimhan, Prasad Deshmukh, Sagar S Gaurkar, Farhat Q Khan, Hellen Y Dzoagbe

**Affiliations:** 1 Otolaryngology-Head and Neck Surgery, Jawaharlal Nehru Medical College, Datta Meghe Institute of Higher Education and Research, Wardha, IND; 2 Pharmacology and Therapeutics, Datta Meghe Institute of Higher Education and Research, Wardha, IND

**Keywords:** vocal fold immobility, voice therapy, laryngeal function, vocal cord paralysis, abductor vocal cord paralysis

## Abstract

Abductor vocal cord paralysis (AVCP) is a disabling disorder that affects the voice and the quality of life of a patient, and hence, there is importance in providing a detailed knowledge of its aetiology and management. In this review article, we offer an implicational definition of AVCP and a discussion of its background, viewed as potentially affecting voice production and health. Finally, we explore modalities of diagnosis by medical history and physical examination, visualization techniques like laryngoscopy and stroboscopy, and radiographic imaging such as computed tomography (CT) and magnetic resonance imaging (MRI) scans. The article reviews and categorizes approaches into operative and non-operative treatments, including injection laryngoplasty, voice therapy, botulinum toxin injections, and the management of Reinke's edema. Surgical approaches, like arytenoid adduction, cordotomy, and posterior cordotomy, are also scrutinized taking their indication, efficacy, and complication profile into consideration. Learning about the advantages and drawbacks of the following experimental yet promising directions like nerve-muscle pedicle implantation, nerve reinnervation, and engineering of tissues is therefore highly necessitated. In conclusion, the review details the measures that have shown to be useful in the treatment process and their impact on the future practice of clinical work, calling for a more clarified structure of the organization of diagnostic, therapeutic, and rehabilitative activities. Future research directions are outlined based on the gaps which include the development of new treatment approaches, the evaluation of treatment for long-term effects, and the need for interdisciplinary cooperation in the medical field for the benefit of the patients.

## Introduction and background

Abductor vocal cord paralysis (AVCP) is a profound neurological condition that is crippling because it involves the posterior cricoarytenoid (PCA) muscle being weak or paralyzed, such that the vocal cords do not abduct (open) very well during in-breathing [1]. This condition can lead to significant respiratory complications, including dyspnea, stridor, and respiratory failure, which can have a profound impact on an individual's quality of life [2]. The prevalence of AVCP is estimated to be around 1.5-2.5 cases per 100,000 people (about the seating capacity of the Los Angeles Memorial Coliseum), with a higher incidence observed in women and individuals over 60 years of age [3]. There are numerous possible reasons for AVCP, ranging from trauma, to tumour, to infections, to iatrogenic injuries [4]. There is no specific test for AVCP. It is diagnosed through clinical observation, laryngoscopy, and electromyography (EMG), all of which can aid in finding the cause and severity of the condition [5]; however, the management of AVCP remains a complex and problematic issue because otolaryngologists, pulmonologists, and speech-language pathologists among others seem to have their own way of looking at the problem [6]. Speech therapy, vocal cord medialization, and reinnervation procedures have all been attempted throughout the years for the treatment of AVCP [7].

Although medical technology and therapeutic approaches have advanced considerably, the treatment of AVCP is still a very complex and involved process [8]. Treatment currently varies from conservative management, i.e., voice therapy and pulmonary rehab, to more invasive intervention surgeries such as arytenoid adduction as well as vocal cord augmentation [9]. Each option has its pros and cons, and the way that it is used is usually dependent on the cause of the paralysis, how severe the symptoms are, and how important it is for that patient [10]. Therefore, even with advancement in medical technology and if many other approaches have been developed, the treatment of AVCP remains a difficult and delicate topic [11].

The aim of this paper is to review and compile all of the literature that is out there on the management of AVCP, to be able to give a complete picture of all of the current treatments. Through a discussion of both conservative and operative measures, this article hopes to illuminate the most effective means of controlling AVCP while, at the same time, shedding light on the areas of ignorance that still linger in this field. Also, we will look into some new treatments and where the research is heading, which will emphasize the need for a combined approach between all disciplines in order to provide the best care for the patient.

## Review

Positions of vocal cords

**Figure 1:**
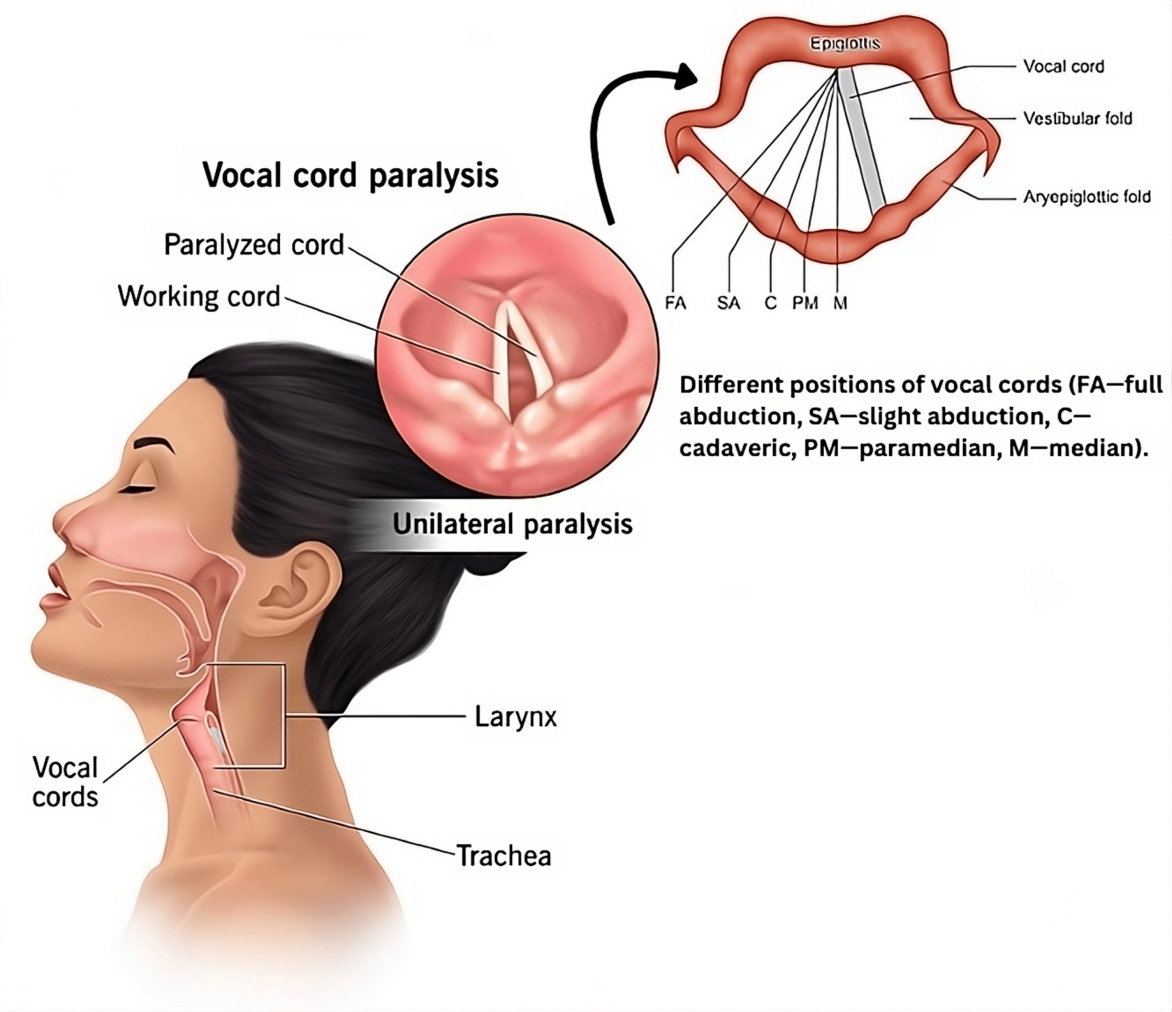
One-sided vocal cord paralysis causes the inhibition of one of the vocal cords from functioning properly, either in opening or in closing FA: full abduction; SA: slight abduction; C: cadaveric; PM: paramedian; M: median Image Credits: Hellen Y. Dzoagbe

The vocal cords, or vocal folds, are two thin, muscular flaps in the larynx, or voice box, at the top of the windpipe [12]. They are held in such a way that when air is blown over them (as in speech or singing), they vibrate and produce sound. The vocal cords are normally in a relaxed state, called the "resting position," and they must stay that way to maintain the health and use of the vocal cords. At rest, the vocal folds are about 17-20 mm long and are angled in a V-shape, with the posterior portion slightly longer than the anterior portion. The V-shape, however, allows for the maximum use of the length of the vocal cords when phonating so that the vocal cords can vibrate effectively and produce a great variety of sounds [13]. In a state of rest, the vocal cords do not touch each other. There is an invention called the rima glottidis, or glottis, which is about 1-2 mm wide. This gap permits air to pass through when breathing normally and keeps the vocal cords from having constant contact, which could cause them to become fatigued and damaged [14]. Figure 1 describes how one-sided vocal cord paralysis causes the inhibition of one of the vocal cords from functioning properly, either in opening or in closing, by one-sided vocal cord paralysis.

**Figure 2:**
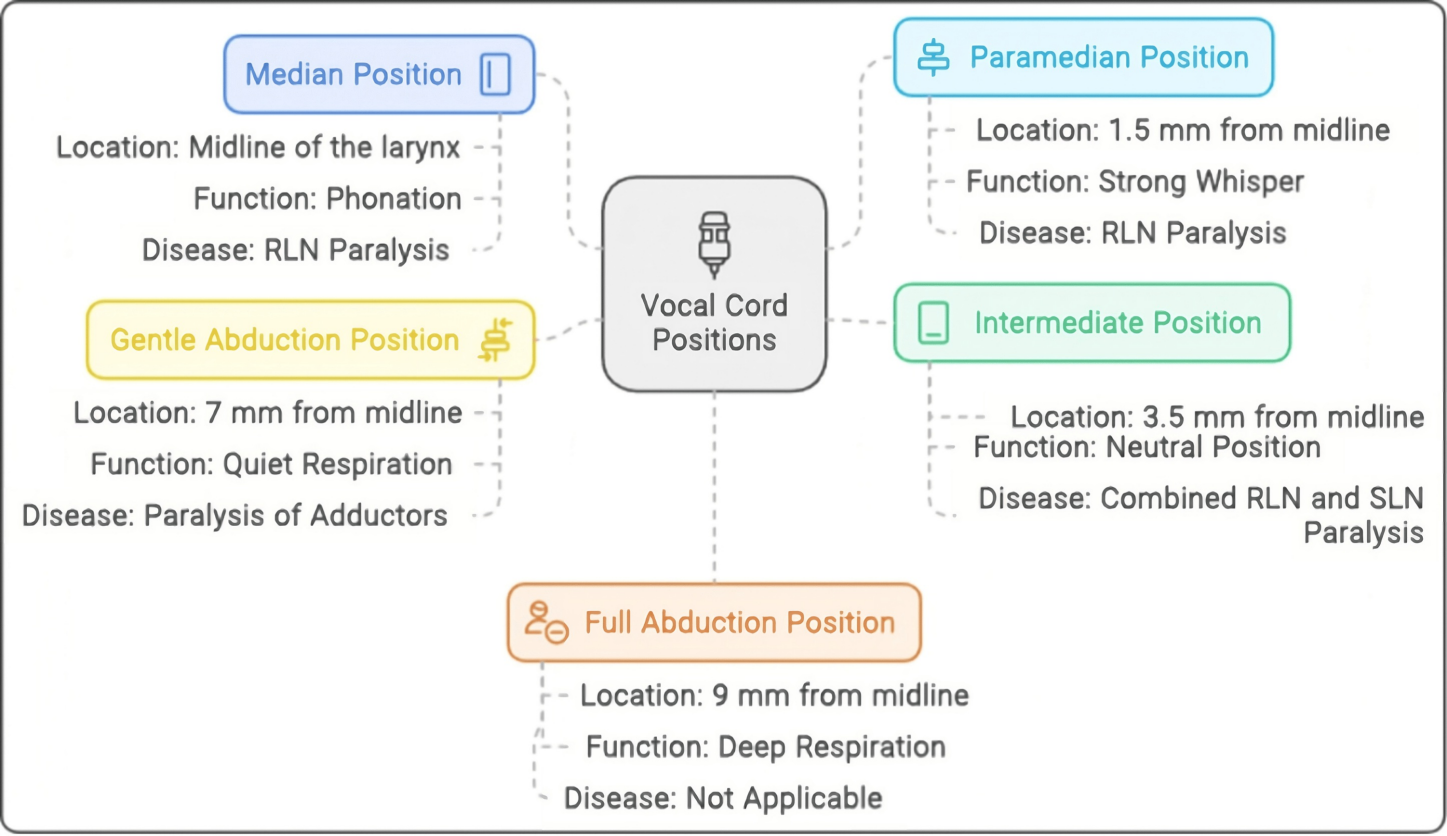
Vocal cord positions RLN: recurrent laryngeal nerve; SLN: superior laryngeal nerve Image Credits: Hellen Y. Dzoagbe

The vocal cords will be in one of the following positions during phonation: The first is in the median position, where the vocal cord is midline, as it would be while phonating. It may occur in recurrent laryngeal nerve (RLN) paralysis [15]. The second is in the paramedian position, where the vocal cord is 1.5 mm away from the midline. It occurs in a strong whisper in a healthy person. It may occur in RLN palsy [16]. The third is in the intermediate (cadaveric) position which is the neutral position of vocal cords. Abduction and addiction occur from this point. The vocal cord lies 3.5 mm away from the midline. This happens with combined paralysis of RLN and superior laryngeal nerve (SLN) [17]. The fourth is in the slight abduction position, where the vocal cord is 7 mm away from the midline. It occurs during quiet respiration and paralysis of adductors [18]. The last is in the full abduction position, where the vocal cord is 9 mm (about 0.35 in) away from the midline, such as in deep respiration [19]. Figure 2 shows the various positions of the vocal cords.

Epidemiology

The epidemiology of vocal cord paralysis was studied extensively in one of the largest studies by Benninger et al. in 1998. They studied the shift in the cause of vocal cord paralysis over a decade from 1985 to 1995. The study included 1,003 patients with vocal cord paralysis, of which 80.3% had unilateral paralysis and 19.7% had bilateral paralysis. The authors found that the most common aetiology for unilateral vocal cord paralysis was malignancy (36%), followed by idiopathic causes (26%) and surgical injury (25%). For bilateral vocal cord paralysis, the most common cause was surgical injury (55.7%), particularly thyroid and parathyroid surgery [20].

AVCP is a serious condition in which the vocal cords cannot abduct, which can cause severe breathing and speaking problems. The epidemiology of AVCP points to surgical trauma as the main culprit, specifically after thyroid and parathyroid surgeries, which comprise a large percentage of adult cases. Congenital causes are more prevalent in children, and bilateral paralysis is especially associated with central nervous system abnormalities [21]. The incidence of bilateral vocal cord paralysis in neonates is estimated at approximately 0.75 cases per million births annually, often coexisting with conditions like prematurity and bronchopulmonary dysplasia. Unilateral vocal cord paralysis is much more common. Still, bilateral cases can result in serious airway compromise and may necessitate interventions such as tracheostomy or surgical procedures to restore airway function. Outcomes range from spontaneous remission, reported in many cases, especially in children, to permanent deficit, underscoring the need for close observation and tailored treatment plans according to aetiology and patient age [22].

Prevalence of vocal cord paralysis

**Figure 3:**
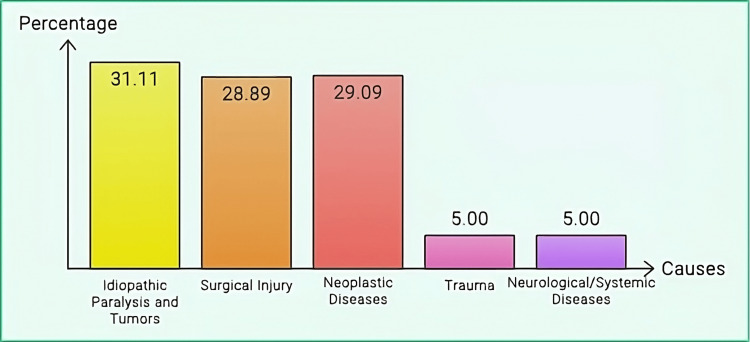
Prevalence of causes of vocal cord paralysis Image Credits: Hellen Y. Dzoagbe

There are several causes of vocal cord paralysis, but they all have different incidences. According to a study, the leading causes of vocal cord paralysis include idiopathic paralysis and tumours, accounting for approximately 31.11% of cases [15]. Surgical injury follows closely behind, responsible for about 28.89% of instances, often resulting from procedures involving the neck, such as thyroid surgery [16]. Additional contributing factors include trauma, though it is not frequently reported, and a range of neurological/systemic diseases, which are less common but still relevant. Another study showed a slightly different distribution, with 29.09% of patients having neoplastic diseases. This further illustrates tumours' significant role in this condition [23]. These facts underscore the multifactorial aetiology of vocal fold paralysis and the need for a comprehensive diagnostic workup to determine the aetiology and direct treatment accordingly. Figure 3 illustrates the prevalence of causes of vocal cord paralysis.

Aetiology of vocal cord paralysis

**Figure 4:**
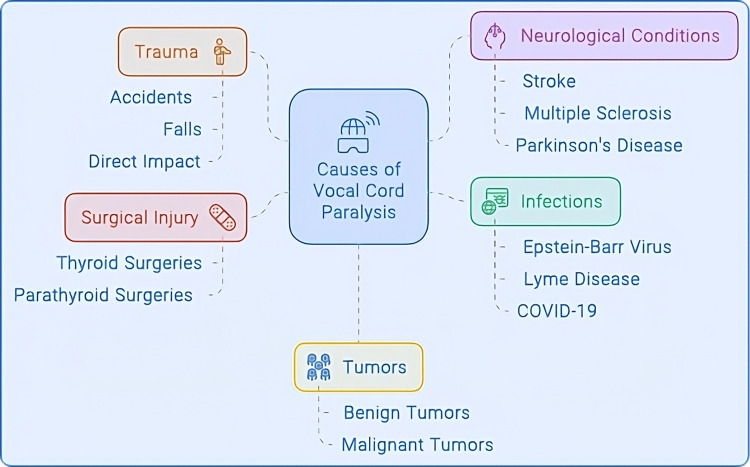
Causes of vocal cord paralysis Image Credits: Hellen Y. Dzoagbe

Vocal cord paralysis occurs when nerve impulses to the larynx become disrupted and one or both vocal cords fail to function properly [24]. This paralysis can interfere with a person's ability to speak, breathe, and swallow and is, therefore, of great clinical concern. The causes of vocal cord paralysis are quite vast and include everything from neurological to mechanical, to infectious, to idiopathic. The aetiology of this condition can be multifactorial. Figure 4 shows some of the causes of vocal cord paralysis.

A study done by Paquette et al. investigated the etiologies of vocal cord paralysis. They found that idiopathic paralysis and tumours made up about 31.11%. That implies that these two pathogeneses are major factors in the incidence of vocal cord paralysis. It also mentioned other reasons, like surgery, trauma, brain pathology, systemic illness, etc., being a cause but not as much [25]. Idiopathic means that there is no known reason for the paralysis, so the doctor has to run many tests to ensure there isn't any underlying cause. Benign and malignant tumours can start in the larynx or the structures immediately next to it. They can impinge on or invade the nerves that control the vocal cords, thus causing paralysis. Surgical injury is another significant contributor, responsible for about 28.89% of vocal cord paralysis cases. The chance of RLN palsy (temporary or permanent paralysis of the vocal cords) is always a risk whenever dealing with procedures around the neck, such as thyroidectomy and other thyroid and parathyroid gland surgery. All the search results make a point that non-thyroid surgery, which has now replaced thyroidectomy, is the most common iatrogenic surgical cause of RLN injury and subsequent vocal cord paralysis [26].

Chiang et al. pointed out that non-thyroid surgeries have become the number one iatrogenic surgical cause of vocal cord paralysis, surpassing thyroidectomy. The change in the number one cause of surgical injury is very important because it shows that more research needs to be done on the risk factors and mechanisms involved in non-thyroid operations that lead to vocal cord paralysis [27]. Phelan et al. showed in a prospective, multicenter study that continuous vagal intraoperative neuromonitoring (IONM) is effective in preventing RLN paralysis because it reveals initial EMG changes of impending neuropraxic injury. This finding suggests that advancements in intraoperative monitoring techniques can be crucial in preventing and managing vocal cord paralysis resulting from surgical injury [28]. Yet, there is a gap in knowledge on the best time to do a postoperative laryngoscopy to diagnose RLN injury. Zakaria et al. and Dionigi et al. conducted studies on RLN injury in thyroid surgery but did not provide specific insights into the timing of postoperative laryngoscopy. Further studies are needed to find the best time for a laryngoscopy after surgery to catch and treat RLN injury as early as possible [23,29].

Adults with congenital bilateral vocal fold immobility (BVFI) are more likely to have an underlying central nervous system disease. An Arnold-Chiari malformation is the most common central nervous system anomaly that causes bilateral vocal fold paralysis. BVFI is also associated with central neurologic degenerative diseases like syringomyelia and syringobulbia [30,31]. Other central nervous system etiologies of vocal fold paralysis include leukodystrophy, encephalocele, hydrocephalus, and cerebral or nuclear dysgenesis [32]. Even peripheral disorders like congenital myasthenia gravis have been associated with congenital BVFI, but that is much more likely to happen in older children and adults [33].

As in adults, surgical trauma can also lead to vocal fold immobility in children. With any surgery so close to the RLN, there is always the chance of vocal fold paralysis. Surgical trauma in neonates typically causes unilateral paralysis; however, patients who undergo tracheoesophageal fistula (TEF) repair, particularly H-type TEF, are predisposed to have bilateral vocal cord paralysis. BVFI can also be caused by blunt trauma and closed head injuries, particularly those that affect the posterior fossa. Other differential considerations for BVFI are metabolic and toxic aetiologies. However, BVFI has also been linked to hypokalemia and organophosphate poisoning [34]. Also, unilateral and bilateral vocal fold paralysis has been reported as a side effect of vincristine with a dose-dependent response which improves slowly upon discontinuation of the drug.

While rare, some genetic syndromes have been associated with familial vocal fold paralysis [35]. Hsu et al. examined the literature and discovered several genetic syndromes linked to BVFI in neonates and adults [36]. An autosomal dominant transmission of neonatal BVFI is associated with a particularly rare balanced translocation between chromosomes 5 and 14 [36]. A different study found 21 cases of familial BVFI and observed different inheritance patterns such as autosomal dominant, X-linked dominant, and X-linked recessive. Other conditions linked with congenital BVFI are 22q11 deletion and Robinow syndromes [37]. Inflammatory processes, underlying infections, and granulomatous diseases have been linked to BVFI. Reported cases of BVFI have been documented in patients with polio, tuberculosis, cytomegalovirus, West Nile virus, and herpes simplex virus [38,39]. On rare occasions, Guillain-Barré syndrome has been associated with BVFI when it affects the larynx, although it rarely affects laryngeal function [37]. Table 1 shows the various etiologies, their description, and their prevalence.

**Table 1:** Aetiology, description, and prevalence of vocal cord paralysis GPCRs: G protein-coupled receptor

References	Aetiology	Description	Prevalence (%)
Cirocchi et al. (2019) [40]	Surgery	Nerve injury with surgery, specifically thyroid, tracheal, and esophageal resections	28.89%
Brown et al. (2017) [41]	Trauma	An injury to the neck or chest, which could crush the nerves of the vocal cords	Not specified
Campbell et al. (2013) [42]	Stroke	When blood flow to the brain is cut off, it can disturb various neural pathways, such as those involved in the control of the vocal cords, which may include GPCRs signaling mechanisms	Not specified
Italiano et al. (2011) [43]	Tumours	Tumours that grow in or around the muscles, cartilages, or nerves of the voice box which may lead to paralysis	31.11%
Yamagishi et al. (2012) [44]	Neurological conditions	Like multiple sclerosis, Parkinson's disease, and myasthenia gravis which can all cause weakness or paralysis of the vocal cords	Not specified
Bhalerao et al. (2015) [45]	Relapsing polychondritis	Inflammatory condition that can lead to the fusion of the vocal cord joints	24.2%
Salik and Winters (2024) [46]	Radiation therapy	Scarring of the arytenoid joints due to radiation therapy	20.9-59%

Diagnostic methods

**Figure 5:**
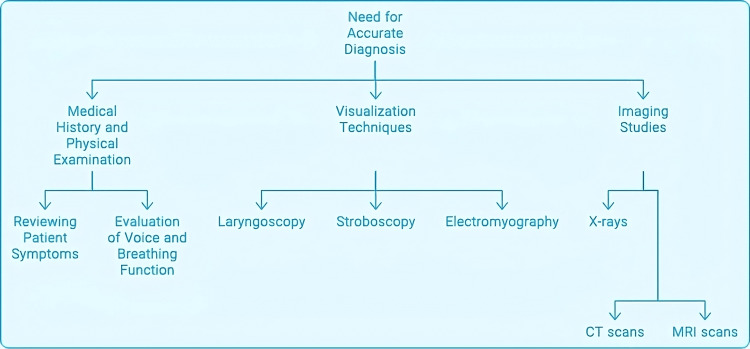
Diagnostic methods for abductor vocal cord paralysis CT: computed tomography; MRI: magnetic resonance imaging Image Credits: Hellen Y. Dzoagbe

The first aspect of diagnosing AVCP involves thoroughly investigating the patient's complaints and past medical history. These symptoms may include a change in their voice, which might become hoarse, breathy, or weakened, or the person may experience some trouble breathing, such as stridor, dyspnea, or the feeling of choking, usually while swallowing. One of the patients' complaints is vocal fatigue, the feeling of being tired after talking or using the voice for a long period [47]. This type of information is usually obtained by healthcare providers through straightforward questioning regarding the time of onset, the length of time the symptoms have been present, and how they have changed over time, as well as any other factors that may have contributed to the dysfunction of the vocal cords, such as a recent infection or surgical procedure. This indicates that breathing and voice assessment is so important in diagnosis. This could include examining the patient's voice quality, pitch, and loudness by completing certain tasks assessing the vocal function, such as sustaining a vowel sound or changing pitch and loudness levels. The clinician will also observe the patient's respirations and may auscultate for abnormal sounds, such as stridor. Diagnosing unilateral v/s bilateral vocal cord paralysis is important because the treatment and management are very different and can be somewhat determined by certain physical findings [48]. Figure 5 shows some diagnostic methods for AVCP.

Medical history and physical examination

A diagnosis of AVCP starts with a thorough patient history (i.e., symptoms and complaints). Patients complain of voice changes, such as hoarseness, breathiness, or a weak voice, because air escapes through the incompletely adducted vocal fold. They might even complain of projection problems, where when they try to speak out, their voice gives out on them or starts to sound weird, and then they feel vocally fatigued all day. Additionally, patients may experience breath-wasting phenomena, where the inability to maintain glottic closure results in a rough voice and breathlessness during speech. Questions about swallowing will come up, complaining about choking or aspirating on oral intake, especially solid things. These symptoms need to be investigated by the clinician to determine whether this is a case of an isolated RLN palsy or a more complicated high vagal lesion, which would present with more severe dysphagia and possibly other symptoms such as nasal regurgitation. A complete history and physical exam and direct larynx examination are necessary for diagnosing and determining the aetiology of vocal cord paralysis [48].

Visualization techniques

Visualization techniques are crucial in diagnosing AVCP because they allow the clinician to observe the movement and functionality of the vocal cords. This is primarily done through laryngoscopy, executed with a rigid or flexible scope. Rigid laryngoscopy provides a clear view of the larynx but is typically conducted under general anaesthesia, while flexible laryngoscopy allows for real-time assessment in an office setting. This allows the clinician to see where the vocal cords rest, how they move while speaking, and if there are any irregularities, such as asymmetry or atrophy [49]. The other very important visualization tool is stroboscopy, which allows for a much better assessment of the vocal folds. It employs a strobe light to create a slow-motion view of the vocal folds during phonation, allowing for detailed analysis of their vibratory patterns. This method is especially good at detecting subtle irregularities in vocal fold movement that would not be noticed in routine laryngoscopy [50].

Besides these methods, there is also laryngeal electromyography (LEMG), which can measure the electrical activity of the laryngeal muscles and thus give information on the status of the RLN and the prognosis for recovery. It is especially helpful in diagnosing the cause of paralysis when the cause is unknown because it can distinguish between a neurogenic and a mechanical cause of vocal fold immobility [51].

Imaging studies

Imaging modalities like computed tomography (CT) and magnetic resonance imaging (MRI) can supplement these visualization methods by offering precise anatomical data regarding the larynx and its adjacent structures [52]. They are very good at visualizing possible causes for vocal cord paralysis, like tumours or structural defects. These imaging modalities combined provide a complete diagnostic approach to AVCP that will allow a definitive diagnosis, and that information will guide future management of the condition [53].

Imaging studies are integral in evaluating AVCP, and CT scanning and MRI are the modalities of choice in these cases. According to Mitchell and coworkers, CT or MRI should be performed in suspected cases of retrosternal extension and fixed tumours (with or without vocal cord paralysis) or when haemoptysis is reported. Paquette and colleagues emphasize the association of CT findings in unilateral vocal cord paralysis with mediastinal aetiologies and the path of the RLN [54]. The guidelines provided by Mitchell et al. emphasize the importance of imaging studies in those cases where local invasion, retrosternal extension, or fixed tumours are suspected. This implies that CT scanning or MRI is especially helpful in determining disease spread and discovering possible aetiologies of vocal cord paralysis in patients with AVCP [54].

Nonetheless, since imaging studies play such a vital role in the workup of AVCP, there appears to be a void in the literature regarding the exact imaging characteristics and findings that are most suggestive of AVCP. While the guidelines by Mitchell et al. and the review by Paquette et al. give some guiding principles as to when to use CT scans and MRIs in the suspicion of AVCP, more exploration is required to find certain imaging characteristics that can diagnose and categorize AVCP. Also, future research should include comparing CT scans to MRI in diagnosing AVCP and the place of more advanced imaging, such as functional imaging modalities, in the workup of AVCP [25,54].

X-rays

Normal laryngeal X-rays can diagnose vocal cord paralysis, but this is not the best method because of superimposed shadows. Dual-energy subtraction (DES) imaging using flat panel detector radiography is a much better modality to evaluate the vocal cords and diagnose paralysis than plain film X-rays, as it has much higher sensitivity and specificity [55].

CT scans

CT from the skull base to the chest should be done in cases of complete vocal cord paralysis to follow the course of the vagus and RLN and discover any lesions that could be causing this. CT can show atrophy of the thyroarytenoid muscle, wasting and tapering of the vocal cord, loss of the subglottic arch, and dilatation of the ventricle and pyriform sinus on the paralyzed side [56].

Paquette et al. reviewed CT findings and mediastinal causes of unilateral vocal cord paralysis in 2012. They investigated the possible mediastinal reasons for vocal cord paralysis and described this abnormality's CT appearance. They stressed that CT imaging should be used to evaluate anatomic structures adjacent to the RLN and search for any mediastinal pathology that could produce vocal cord paralysis. This research has shed light on using CT scans as a diagnostic tool for vocal cord paralysis and the importance of imaging methods in determining mediastinal pathology [25].

Song et al. performed a 10-year retrospective study using CT imaging to assess the vocal cord paralysis caused by thoracic diseases. They studied to find out which thoracic diseases caused vocal cord paralysis and what the CT findings would be with this aetiology. They stressed that CT analysis is crucial in determining thoracic diseases that could lead to a paralyzed vocal cord. Their study adds to the knowledge of thoracic disease and vocal cord paralysis and shows how CT imaging can diagnose this problem when thoracic pathologies are present [57].

MRI

MRI of the brain is recommended to exclude intracranial pathologies like multiple sclerosis or mass lesions, especially when no cause is identified on initial evaluation or when vagal symptoms are present. An MRI is a substitute for CT but has a much lesser chance of false positives [58]. AVCP is a condition that must be diagnosed correctly to understand its aetiology and thus be treated correctly. MRI or CT is indicated if the retrosternal extension is suspected, the tumour is fixed, or haemoptysis is present [54]. This is very important because CT has proved useful in detecting primary malignancy or the progression of malignancy between follow-ups. Song et al. stated that CT is especially helpful in evaluating vocal cord paralysis from thoracic disease, giving important information for the diagnosis and follow-up of this condition [57].

Another example comes from the use of LEMG in diagnosing and treating vocal cord paralysis, in which recovery can be predicted based on motor unit potential recruitment and the presence of polyphasic motor unit potentials within the first six months after lesion onset [59]. This shows that EMG is a crucial component in imaging studies and must be used to evaluate the functional aspects of vocal cord paralysis and its recovery potential [60]. Although the current body of research offers many interesting theories, there is still a lack of understanding of the diagnostic techniques for AVCP. Future research should involve a comparative study between MRI and CT scans in diagnosing and following up vocal cord paralysis, with sensitivity, specificity, and cost-effectiveness in mind. Also, more research is needed on combining EMG with imaging studies to increase the diagnostic and prognostic value, which would, in turn, lead to a better approach to diagnosing vocal cord paralysis.

Management of vocal cord paralysis

AVCP is not a simple condition; its treatment is not simple. It depends on many factors like aetiology, symptoms, and the duration of paralysis, among many others. Conservative measures, surgical interventions, and supportive therapies are all a part of the AVCP management.

If conservative management does not provide a satisfactory outcome or if the paralysis is severe, surgical intervention is a possibility. Bulk injection involves adding volume to the paralyzed vocal cord by injecting collagen, hyaluronic acid, or autologous fat. This procedure aims to pull the paralyzed cord toward the midline so that when the good cord vibrates (during phonation and swallowing), it is more likely to make better contact. Another is the surgical approach of vocal cord repositioning, which involves using a piece of the patient's tissue to push the paralyzed vocal cord back toward the midline, thus allowing the functioning vocal cord to vibrate better against the paralyzed one. This procedure is called a thyroplasty or medialization laryngoplasty, and it places an implant into the larynx to push the vocal cord back into position. It can give more permanent results. If the nerve injury is severe enough, reinnervation may be an option. This procedure involves taking a healthy nerve from somewhere else in the body and transplanting it to the damaged area, where it will eventually allow for better function [61].

If the bilateral vocal cord paralysis is severe enough to cause airway obstruction, then a tracheotomy may be required. The surgical procedure opens up the trachea so air can pass through the vocal cords and provide enough ventilation. A tracheotomy is usually a desperate move and is only done in an emergency. New treatments for AVCP may include electrical stimulation, which would entail connecting the vocal cords to some electrical stimulation device to get them to move again, and gene therapy, which might look at genetic modifications to help the nerves regenerate and work better. These therapies are still in the investigational stage [62].

Supportive care is a major aspect for patients with AVCP. This may include nutritional support via a feeding tube for those who have difficulty swallowing and psychological support for the emotional impact of the disease. Otolaryngologists and speech-language pathologists should follow up regularly to track progress, to make necessary changes to the plan of care, as well as to identify new problems that may arise [58]. The outlook for AVCP is very different for each patient, depending on the cause of the paralysis, how severe it is, and how soon it is treated. Most individuals with unilateral paralysis will recover significantly over time, especially with proper therapy. Still, those who have bilateral paralysis will be faced with much more difficult challenges and will need continual management.

Treatment

**Figure 6:**
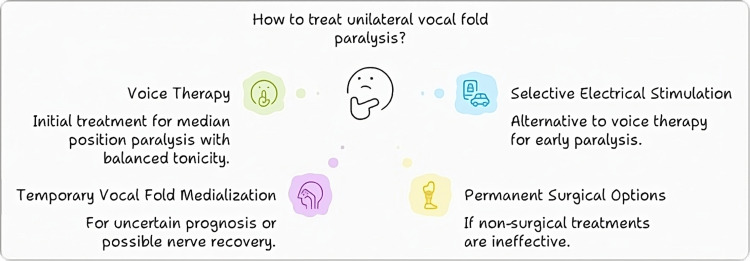
Treatment approaches for abductor vocal cord paralysis Image Credits: Hellen Y. Dzoagbe

The treatments for AVCP began in the mid-19th century with the work of Manuel Garcia, who invented mirror laryngoscopy in 1855, which provided a better view of the larynx [63]. Chevalier Jackson performed the first surgical intervention for BVFI in 1922, who endoscopically resected a vocal cord to improve airway management at the expense of voice quality, highlighting a persistent dilemma in laryngology between maintaining voice function and ensuring adequate airway protection [64]. Throughout the years, many different surgical approaches have been developed, ranging from adenoidectomy to vocal cord lateralization, and with the more modern laser surgery, the prognosis and complications have been improved. The introduction of bulk injections to add volume to paralyzed vocal cords emerged as a significant innovation, allowing for better approximation between the vocal cords during phonation [65]. Within the last decade, experimental methods such as reinnervation procedures and electrical pacing have been explored to recover vocal cord movement via nerve regeneration or muscle stimulation. This historical development is just a constant struggle between airway safety and voice quality, constantly changing with the new surgical techniques and technology that becomes available, as well as the aetiologies of the paralysis itself, be they neurogenic, traumatic, inflammatory, or iatrogenic [46]. Figure 6 depicts the treatment approaches for AVCP.

Voice therapy

Voice therapy is vital for AVCP, especially after thyroid surgery. Some research suggests that voice therapy involving muscle relaxation, breathing exercises, and some more specialized phonation training can help patients who have unilateral vocal cord paralysis occurring as a result of thyroid surgery regain their voice [66]. Bilateral AVCP has been somewhat remedied by the surgical techniques of vocal cord lateral fixation and endo-extra laryngeal suture lateralization, which restore both vocal cord tone and abductor movements without requiring a tracheostomy [67]. Even better, they propose selective reinnervation of the larynx, giving back vocal cord tone and abductor movements to those suffering from bilateral vocal cord palsy [68]. In patients with bilateral AVCP where the compromised airway could be life-threatening, CO2 laser-assisted posterior cordotomy and collator-assisted posterior cordectomy are safe and efficacious methods of dealing with airway compromise caused by paralysis of the vocal folds [69]. Also, for the symptomatic patient with laryngeal abductor dysfunction, noninvasive continuous positive air pressure during sleep or surgical options such as vocal cord lateralization, cordectomy, or tracheotomy should be considered because this condition can be fatal if left alone [70].

Mastering the pathophysiology behind AVCP is a must for voice therapy to be successful. Abductor muscles are paralyzed, so one can't start phonation or talk in a normal voice. There are many different causes of paralysis, ranging from traumatic injury to neurological disorders to iatrogenic causes. AVCP voice therapy is designed to remediate the basic impairments in vocal fold movement, coordination, and respiration [71].

AVCP is a rare and crippling disease that robs one of the abilities to speak, which can be very emotionally, socially, and professionally distressing. The main purpose of voice therapy for AVCP is to restore vocal function, relieve symptoms, and improve quality of life. Voice therapy is essential to this disorder's overall medical, surgical, and behavioural treatment plan. Many voice therapy techniques have been designed to treat the deficits that come along with AVCP. One of the most promising methods is the Lee Silverman voice treatment (LSVT) approach, which aims to increase vocal fold abduction and vocal quality and amplify vocal loudness. The other method, vocal function exercises (VFE), focuses on building up the vocal folds, vocal fold closure, and vocal control. Also, visual feedback like video stroboscopy can help with better vocal fold function and coordination [72]. Numerous studies have shown that voice therapy improves vocal function and overall quality of life in those who suffer from AVCP. According to a study in the Journal of Voice, 80% of patients with AVCP have shown great improvement in vocal function with voice therapy [73]. According to another article in the International Journal of Language & Communication Disorders, voice therapy led to better vocal quality, greater vocal volume, and improved psychological state in those with AVCP [74]. 

Surgical treatments

There are several surgical options for AVCP, such as arytenoidectomy, posterior cordectomy, and reinnervation [75]. Also, endoscopic/assisted microscopic posterior cordotomy with the use of radiofrequency or monopolar microelectrodes has proven to be beneficial to those suffering from bilateral abductor vocal fold paralysis in that it improves the postoperative glottic chink, exercise tolerance, dyspnea, and aspiration [76]. These surgical procedures are so important because they ensure an open airway, maintain voice quality, and help promote laryngeal closure (which is a big problem with AVCP patients), allowing these patients to breathe better, talk better, eat better, and live better.

Benjamin et al. performed a retrospective case study review to examine the medical outcomes of patients with acute unilateral vocal cord paralysis who underwent percutaneous injection laryngoplasty with bovine collagen and local topical anaesthesia. The study found that the procedure significantly improved vocal function, as assessed by the glottal function index (GFI), grade, roughness, breathiness, asthenia, strain (GRBAS) dysphonia scale, functional outcome swallowing scale (FOSS), and maximum phonation time (MPT). Also, the researchers found no problems associated with the procedure; therefore, injection laryngoplasty is a viable option for primary rehabilitation in an acute setting. However, when it comes to patients who had dysphagia and multiple cranial neuropathies, success was not as great in restoring oral alimentation. This research suggests that percutaneous injection laryngoplasty is a suitable alternative for the immediate rehabilitation of vocal fold paralysis and its importance lies in improving patient care in acute care facilities [77].

In the study by Røksund et al. in 2010, the authors found a novel clinical situation with left vocal cord paralysis in adults born extremely preterm. This study highlighted the lasting effects of vocal cord paralysis in preemies, showing that although the condition may seem to go unnoticed for many years, it can greatly affect vocal ability and quality of life when these preemies become adults. Yes, the research stressed the necessity for continual observation of vocal cord dysfunction in these patients because if caught in the early stages or surgically corrected, it could do wonders for their symptoms and prognosis. On the whole, the results emphasize the importance of increased consciousness and aggressive medical management in the adult population with a history of extreme prematurity, especially concerning their voice [61].

Graboyes et al. investigated the effectiveness and safety of acute injection laryngoplasty in treating vocal cord paralysis after thoracic surgery. This retrospective analysis involved 20 patients and aimed to evaluate the immediate postoperative outcomes of this surgical intervention. The findings revealed that injection laryngoplasty significantly improved vocal function and had a favourable safety profile with no reported complications. Not only that, but the research also showed that this procedure lowered the likelihood of needing more laryngeal procedures, which proves that this is a good treatment for those patients who suffer from vocal cord paralysis in the acute setting. In conclusion, the study provides significant findings regarding using injection laryngoplasty to treat vocal cord dysfunction. It underscores its ability to improve the prognosis of those undergoing thoracic surgery [78].

Surgical management of AVCP is a broad field of study, with many avenues of research, such as the efficacy and safety of injection laryngoplasty, long-term morbidities associated with vocal cord paralysis, and special considerations for pediatric patients and complex surgical cases. Although this prior research does shed light on many aspects of this topic, there are still unknowns as to the best surgical approaches, what the long-term results will be like, and what other research should be conducted in the future. Further research should aim to fill in these holes, perhaps concentrating on perfecting surgical techniques, bettering patient prognosis, and expanding knowledge of vocal cord paralysis in different patients.

Novel and experimental treatments

Experimental treatments for AVCP are being developed in hopes of restoring vocal function and airway control to those suffering from this disorder. A very promising procedure is laryngeal reinnervation, which is the transplant of a healthy nerve into the paralyzed vocal cord, and there are some great results with voice and swallowing. Furthermore, laryngeal pacing uses electrical stimulation to make the vocal cords move and is being tested in some clinical trials, and it seems to show some promise in increasing vocal cord movement. Other experimental therapies include botulinum toxin injections and bulking agents like collagen or hyaluronic acid to plump up the vocal cords, but these are still being researched [47].

Also, transoral injections of substances such as Cymetra have shown preliminary success in enhancing voice quality, but the results are not likely to be permanent. A study by Karpenko et al. studied the efficacy of transoral injection of Cymetra in 10 patients suffering from breathy dysphonia due to unilateral vocal fold paralysis. The researchers observed significant increases in MPT, relative glottal area, and subjective global rating of glottal competency right after the procedure. However, these improvements were not sustained at the three-month follow-up, and no statistically significant difference in objective or subjective voice quality was found in the study. The authors believe that the resorption of Cymetra plays a large part in these findings [79].

The other experimental method is PCA reinnervation and pacing for bilateral vocal fold paralysis. Broniatowski et al. tested whether the respiratory distress caused by bilateral vocal fold paralysis could be quantifiably relieved by this method. They also had a case in which a patient with paramedian vocal folds and synkinesis had a tracheotomy for stridor due to bilateral laryngeal nerve injury as a result of Miller Fisher syndrome. One PCA received a nerve-muscle pedicle with a perineural electrode for pacemaker stimulation. The airway was assessed by endoscopy and spirometry for up to one year. They discovered bilateral vocal fold patency was reversed during normal breathing to active, vocal fold adduction when the trachea was occluded. Peak inspiratory flows were much greater after reinnervation and even more so during stimulation, but these differences were insignificant. From these initial data, the authors surmised that PCA reinnervation and pacing may hold hope for improving respiratory impairment following paradoxical adduction in bilateral vocal fold paralysis [80].

Another novel approach is to use structural implants (thyroplasty, medialization laryngoplasty, laryngeal framework surgery) to shift the vocal cord back into place. Here, a small implant is inserted into the larynx, pulling the paralyzed vocal cord into the middle of the voice box. This causes the healthy vocal cord to meet the paralyzed one better when talking, swallowing, or coughing. Although very seldom, sometimes with this kind of surgery, the person has to return for a second surgery to have the implant shifted into a different position [78].

According to Trozzi et al., endoscopic arytenoid lateral abduction (EALA) is a safe and efficacious method for treating pediatric BVFI. The decannulation rate in patients with tracheostomy was 91%, with decannulation occurring at 4.4 weeks on average. However, in non-trachea patients, there was a significant improvement in peak inspiratory flow and number of desaturations/hr postoperatively. However, one month after surgery, the pediatric voice handicap index, GIRBAS (grade, instability, roughness, breathiness, asthenia, strain) scale, and Montreal Children's Hospital feeding scale all showed deteriorated scores, which, by one-year follow-up, were similar to their preoperative scores (except for the B and A parameters of the GIRBAS score) [81]. The field of novel and experimental treatments for AVCP is rapidly changing, and there is much hope for this condition. Although some of these methodologies have successfully enhanced voice capabilities and airway control, more long-term studies are required to determine their effectiveness and safety. As these methods are still being perfected and tested in clinical trials, hopefully they will someday become more accessible and soon become part of the standard treatment regimen for vocal cord paralysis patients.

## Conclusions

AVCP is a terrible ailment that seriously affects a person's life. Many different factors cause AVCP, the three most common being surgical trauma, idiopathic causes, and tumours. The diagnostic workup includes a complete medical history, physical examination, and visualization techniques such as laryngoscopy and stroboscopy. Imaging studies such as CT and MRI scans also reveal underlying lesions or structural abnormalities. The management of AVCP is a complicated process that demands a multidisciplinary approach. Voice therapy is a key element to better vocal function and alleviation of symptoms. Surgical options such as adenoidectomy, posterior cordectomy, and reinnervation procedures can all restore vocal cord motion and airway control. New and unexplored methods like laryngeal pacing and structural implants may improve voice quality and airway opening. Still, more investigation is required to create better and more lasting treatments for AVCP. The evolution of new surgical methods, the examination of long-term results, and the research of novel treatment modalities are all imperative for advancing the prognosis and quality of life of those afflicted with AVCP. Having a complete knowledge of the cause, diagnosis, and treatment of AVCP will allow healthcare professionals to give the best possible care and management to these patients.
